# A Silent Epidemic of Congenital Anomalies and Its Predictors Among Newborns in Ethiopia: A Systematic Review and Meta-Analysis

**DOI:** 10.3389/phrs.2026.1608833

**Published:** 2026-02-23

**Authors:** Mitku Mammo Taderegew, Alemayehu Wondie, Feredegn Talargia Belete, Wondosen Debebe, Lemlemu Maru, Gashaw Garedew Woldeamanuel

**Affiliations:** 1 Department of Biomedical Sciences, Asrat Woldeyes Health Science Campus, Debre Berhan University, Debre Berhan, Ethiopia; 2 Department of Biomedical Sciences, College of Medicine and Health Sciences, Wolkite University, Wolkite, Ethiopia; 3 Department of Biomedical Sciences, College of Medicine and Health Sciences, Wollo University, Wollo, Ethiopia; 4 Department of Human Physiology, School of Medicine, College of Medicine and Health Science, University of Gondar, Gondar, Ethiopia

**Keywords:** congenital anomalies, prevalence, predictors, newborns, meta-analysis, Ethiopia

## Abstract

**Objective:**

This review was conducted with the aim of assessing the pooled prevalence of congenital anomalies and its predictors among newborns in Ethiopia.

**Methods:**

The relevant studies were identified by electronic database searching methods. All statistical analyses were carried out with STATA™ Version-14 software.

**Results:**

A total of 22 studies were included in this review. The pooled prevalence of congenital anomalies was 21.25 per 1,000 newborns. Age of the mother 35 years and above (POR = 3.29, 95% CI: 1.59–6.82) absence of formal education among mothers (POR = 1.35, 95% CI:1.12–1.63), maternal cigarate smoking (POR = 2.98, 95% CI:1.56–5.67), alcoholic drinking (POR = 2.66, 95% CI:1.28–5.51), chat chewing (POR = 3.37, 95% CI:1.57–7.21), no folic acid supplementation (POR = 4.29, 95% CI:2.35–7.83), pesticide exposure (POR = 3.23, 95% CI: 2.02–5.16), medication use during pregnancy (POR = 4.37, 95% CI:2.11–9.06), maternal chronic illness (POR = 3.76, 95% CI:1.72–8.20), preterm (POR = 2.35, 95% CI: 1.59–3.47), multiple pregnancy (POR = 3.66, 95% CI:1.99–6.71), low birth weight (POR = 5.46, 95% CI: 4.41–6.75) were identified as the predictors of congenital anomalies among newborns in Ethiopia.

**Conclusion:**

Relatively high burden of congenital anomalies were detected. Hence, strategies to minimize substance use, exposure to pesticide and medication, and to improve folic acid supplementation during pregnancy should be encouraged.

## Introduction

Congenital anomaly is a structural or a functional fault that occurs during intrauterine life and may be noticed at the time of intrauterine life or observable at delivery or later in infancy. It comprises visible and microscopic defects, inborn errors of metabolism, intellectual disability, and molecular aberrations. Congenital anomalies begin to emerge as a challenging health problem, representing a major cause of infants’ morbidity, long term disability, and mortality all over the world especially in developing countries [1, 2].

Globally, an estimated 8 million infants each year, representing approximately 6% of all live births, are born with congenital anomalies [2, 3]. Moreover, substantial portion of infants with congenital anomalies die in the first month of life. According to the World Health Organization (WHO) 2023 reports, approximately 240,000 newborns die annually within the first 28 days due to these congenital anomalies. Furthermore, management and curing of children with congenital anomalies are costly and complete recovery may be impossible. The burden is particularly high in low- and middle-income countries as 94% of sever congenital anomalies occurring in these regions [4–6].

According to Health Newborn Network (HNN) data in Sub-Saharan Africa, 71300 neonates were died in the year 2021 due to congenital anomalies, which account for approximately 13% of neonatal deaths, making them a significant contributor to infant mortality in the region. HNN 2021 reported results also revealed that congenital anomalies are among the leading cause of neonatal mortality in Ethiopia which accounts for 5.5% of neonatal death [7].

The proportion of perinatal deaths attributed to congenital anomalies has been increasing, primarily due to a decline in mortality from other causes such as prematurity, birth asphyxia, and neonatal sepsis. This shift is largely the result of advancements in prenatal and neonatal healthcare that have improved outcomes for these conditions. Consequently, congenital anomalies are projected to become a leading cause of infant morbidity and mortality in the coming decades [8].

Despite these devastating effects of congenital anomalies, limited studies had tried to assess the magnitude of congenital anomalies and its predictors among newborns in Ethiopia. In addition, most of these limited studies were conducted at single centers, involved relatively small sample sizes, and exhibited considerable variability and inconsistency in their findings. Accordingly, this systematic review and meta-analysis aimed to determine the pooled prevalence of congenital anomalies and predictors among newborns in Ethiopia by synthesizing findings from existing studies. Therefore, the findings of this study can provide essential baseline information for policymakers and relevant stakeholders to guide the planning and implementation of effective prevention and intervention strategies against congenital anomalies. The results of the study may also serve as foundational data for researchers interested in this field to conduct further investigations.

## Methods

### Protocol, Search Strategy, and Study Selection

A systematic review and meta-analysis was conducted to evaluate the pooled prevalence of congenital anomalies among newborns in Ethiopia. The review was conducted in accordance with the guideline of the Preferred Reporting Items for Systematic Reviews and Meta-Analysis (PRISMA) checklist [9]. The review was registered in the International Prospective Register of Systematic Reviews (PROSPERO) 2024 CRD42023426258 Available from https://www.crd.york.ac.uk/PROSPERO/view/CRD42023426258.

The literature search was conducted across multiple databases, including PubMed, Scopus, Google Scholar, Web of Science, and Wiley Online Library. During exploration of the available study the following key search terms and Medical Subject Headings [MeSH] “Prevalence”, “Magnitude”, “Congenital Abnormalities”, “Congenital Malformation” “Congenital Anomalies” “Birth defects” “Associated Factors”, “Predictors”, “Determinant”, “Newborns” “Infants” and “Ethiopia” were used separately or in combination with the Boolean operator’s terms “AND” and “OR” (Supplementary File 1). Additionally, the reference lists of all retrieved articles were examined to find more relevant studies. The search incorporated studies available up to the 28th of February 2025. All identified articles were imported into EndNote version X20, and duplicate records were removed. Subsequently, a comprehensive screening process was conducted, including evaluation of titles, abstracts, and full-texts, as well as an assessment of article quality based on predefined eligibility criteria. Finally, the eligible studies were compiled together for analysis.

Inclusion and exclusion criteria: All primary studies that reported the prevalence of congenital anomalies and/or its predictors among newborns in Ethiopia were illegible for the study. However, the following studies were excluded: those that were not fully accessible despite two attempts to contact the corresponding author via email or if the outcome of interest could not be determined or calculated based on the available data; and studies that received a poor quality score according to the predefined criteria.

### Data Extraction and Quality Assessment

The selected studies were carefully examined, and the essential data was independently extracted and summarized by two authors using a standardized data extraction spreadsheet format created in Microsoft Excel. If any disagreements arose between the data extractors, a third author was consulted to resolve them. For each included study, information was extracted with regard to the corresponding author, publication year, region of the study, study design, sample size, the magnitude of congenital anomalies, and identified predictors. Two-by-two data were also extracted for each identified factors.

The methodological quality of each included study was independently assessed by two authors using the Newcastle-Ottawa Scale (NOS), adapted for assessing the quality of observational studies [10]. Any disagreements between reviewers regarding the quality assessment of individual articles were resolved through discussion among all authors, with the final decision reached by consensus. Ultimately, only studies that scored 5 or more out of 10 across the three domains of the modified NOS were included in the analysis.

In addition, three authors independently evaluated the risk of bias in the included studies using the risk of bias tool designed for prevalence studies developed by Hoy et al. [11]. The assessment tool included ten criteria, with each unmet criterion scored as 0 (no). The total score was calculated by summing the responses, yielding an overall quality score between 0 and 10. Any discrepancies at the time of data abstraction were fixed by discussion and consensus. When available data were insufficient to make a judgment on a specific criterion, the corresponding authors were contacted for clarification. If uncertainty persists, the item was scored as 0, indicating a high risk of bias. Eventually, studies receiving 8 or more ‘yes’ responses were classified as having a low risk of bias, those with 5–7 ‘yes’ responses as moderate risk, and those with 4 or fewer ‘yes’ responses as high risk.

### Data Processing and Analysis

Heterogeneity among the included studies was assessed using the I^2^ statistic. The results indicated substantial heterogeneity (I^2^ = 99.8%; P < 0.001). Therefore, a random-effects model was used to estimate the pooled prevalence. The pooled prevalence, together with its corresponding 95% confidence interval (CI), was calculated and displayed using a forest plot. Potential sources of heterogeneity were further explored using subgroup analyses based on study region, year of publication, sample size, and risk of bias. Furthermore, meta-regression analyses were performed using year of publication and sample size as covariates to examine their contribution to heterogeneity. To evaluate the influence of each study on the overall assessment, a sensitivity analysis was performed by systematically excluding each study one at a time. Additionally, publication bias was assessed using funnel plot symmetry, Egger’s regression test, and Begg’s test. Finally, the various predictors were expressed using pooled odds ratios (PORs) with corresponding 95% CI. Heterogeneity tests, publication bias, and sensitivity analysis also carried out for each identified predictors accordingly. All statistical analyses were performed using STATA™ version 14 software (StataCorp LP, College Station, TX, USA).

## Results

### Selection of the Studies

**FIGURE 1:**
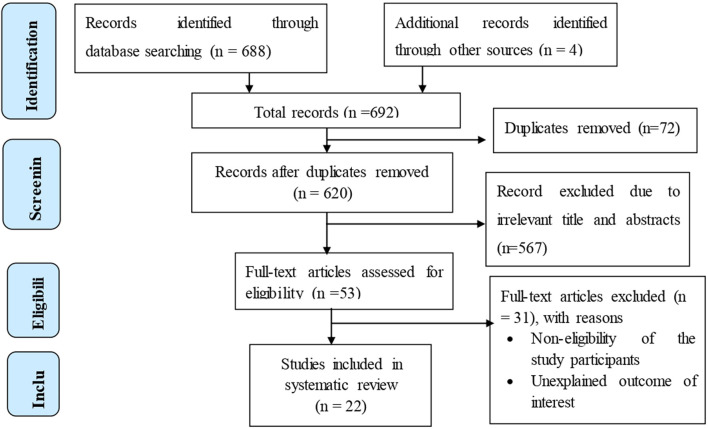
Flow chart showing the selection of studies for the systematic review and meta-analysis of congenital anomalies and its predictors (Ethiopia, 2025).

A total of 692 records regarding the prevalence of congenital anomalies and its predictors among newborns in Ethiopia were retrieved using electronic database searches. Of the identified records, 72 studies were excluded due to duplication. Following title and abstract screening, an additional 567 studies were excluded as they were not relevant for this systematic review and meta-analysis. The remaining 53 full-text articles were assessed for eligibility based on the predefined criteria, resulting in further exclusion of 31 studies. Ultimately, 22 studies that met all eligibility requirements were included in the final systematic review and meta-analysis (Figure 1).

### Baseline Characteristics of Included Studies

In this study total of 22 original studies published between 2013 and 2025 years that showed the magnitude of congenital anomalies and/or its predictors among newborns in Ethiopia were included. The sample size of the included studies ranged from 295 in North Wollo Zone [12] to 45951 in Southwest Ethiopia [13]. The highest prevalence of congenital anomalies was stated in the study conducted in Butajira Hospital 57.5 per 1,000 birth [14] while the lowest was stated in the study conducted in Kersa district Eastern Ethiopia 3.8 per 1,000 [15] (Table 1).

**TABLE 1:** Characteristics of studies reporting congenital anomalies and its predictors among newborns (Ethiopia, 2025).

Author/s (publication years)	Publication years	Region	Study design	Sample size	No of cases	Prevalence per 1,000 (95% CI)	Quality of score (10 pts)	Overall risk of bias
Getachew et al. [16]	2023	Oromia	IBCS	754	31	41.1 (37.59, 44.61)	8	Low risk
Adane and Seyoum [17]	2018	Amhara	IBCS	19,650	317	16.1 (15.59, 16.61)	7	Low risk
Gedamu et al. [18]	2021	Oromia	IBCS	2,218	23	10.4 (9.13, 11.67)	8	Moderate risk
Geneti et al. [13]	2021	Oromia	IBCS	45,951	253	5.5 (5.29, 5.71)	7	Moderate risk
Mekonen et al. [19]	2015	Tigray	IBCS	1,516	32	21.1 (19.05, 23.15)	9	Moderate risk
Mekonen et al. [20]	2021	Tigray	IBCS	12,225	383	31.3 (30.48, 32.12)	7	Low risk
Mekonnen et al. [21]	2021	Amhara	IBCS	11,177	69	6.2 (5.75, 6.65)	7	Moderate risk
Tsegaye and Kassa [22]	2018	SNNPR	IBCS	580	6	10.3 (7.83, 12.77)	8	Moderate risk
Abdu and Seyoum [23]	2019	Amhara	IBCS	22,624	324	14.3 (13.84, 14.76)	7	Moderate risk
Cherie and Mebratu [24]	2017	Amhara	IBCS	462	17	36.9 (32.50, 41.30)	8	Moderate risk
Abdo et al. [14]	2019	SNNPR	IBCS	313	18	57.5 (52.02, 62.98)	9	Low risk
Alemayehu et al. [25]	2022	Amhara	IBCS	371	11	29.7 (24.95, 34.25)	9	Low risk
Eshete et al. [12]	2013	Amhara	IBCS	295	3	10.2 (6.75, 13.65)	6	Moderate risk
Abdo et al. [26]	2016	SNNPR	IBCS	327	6	18.3 (14.11, 22.49)	7	Moderate risk
Mekonnen et al. [27]	2018	Somalia	IBCS	1,050	15	14.3 (12.18, 16.42)	8	Low risk
Degno et al. [28]	2021	Oromia	IBCS	576	18	31.3 (27.51, 35.09)	6	Moderate risk
Didisa MK, et al. [15]	2025	Oromia	IBCS	27,350	104	3.8 (3.57, 4.03)	8	Low risk
Mekonnen et al. [29]	2020	Oromia	Case-control	409	136	----	9	Low risk
Abebe et al. [30]	2021	Oromia	Case-control	1,138	251	----	9	Moderate risk
Jemal et al. [31]	2021	Oromia	Case-control	418	105	----	7	Moderate risk
Tsehay et al. [32]	2019	Amhara	Case-control	398	100	----	7	Moderate risk
Demelash et al. [33]	Unpublished	Harar	IBCS	1,112	36	32.40 (29.65, 35.15)	7	Low risk

IBCS, Institution based cross-sectional study.

### Prevalence of Congenital Anomalies Among Newborns in Ethiopia

**FIGURE 2:**
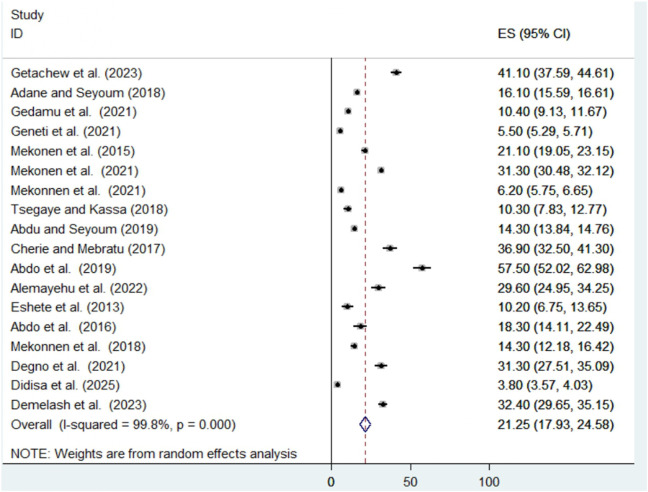
Forest plot of the pooled prevalence of congenital anomalies among newborns (Ethiopia, 2025).

The pooled prevalence of congenital anomalies among newborns was estimated to be 21.25 per 1,000 (95% CI: 17.93–24.58) with a significant level of heterogeneity as evidenced by I^2^ statistic (I^2^ = 99.8%; p < 0.001). This finding indicates a great variability in the prevalence of congenital anomalies among newborns across the included studies. Hence, a random-effects model was employed to estimate the pooled prevalence (Figure 2).

### Subgroup and Meta-Regression Analysis

To identify the possible source of heterogeneity across the included studies, subgroup analysis based on the region where the study was conducted, years of publication, and sample size were conducted. Although only one study done in Harari and Somali region with prevalence of 32.40% and 14.30%, respectively, sub- group analysis shows the highest prevalence was observed in Southern Nation Nationality and People Region (SNNPR) with prevalence of 28.6% followed by Tigray region 26.25% while the lowest prevalence was observed in Addis Ababa 16.33%. Subgroup analysis also revealed that the pooled prevalence of congenital anomalies was higher in studies with a sample size smaller than the median of the total sample size (<1,081) compared to those with a sample size equal to or greater than the median (27.61% vs. 15.58%) (Table 2). Additionally, a meta-regression analysis was performed using year of publication and sample size as covariate. However, none of these variables were statistically significant for the presence of heterogeneity.

**TABLE 2:** Subgroup analysis of the prevalence of congenital anomalies among newborns (Ethiopia, 2025).

Sub-group	Category	Number of studies	Sample size	Prevalence (95% CI)	Heterogeneity	P-value	I^2^ (%)
Region	Oromia	5	76,849	16.33 (13.50, 19.16)	789.15	0.000	99.5
	Amhara	6	54,519	18.32 (13.59, 23.05)	1,167.8	0.000	99.7
	SNNPR	3	1,220	28.60 (4.05, 53.15)	266.9	0.000	99.2
	Tigray	2	13,741	26.25 (16.25, 36.24)	81.6	0.000	98.8
	Somali	1	1,050	14.30 (12.18, 16.42)	0.00	-	-
	Harari	1	1,112	32.40 (29.65, 35.15)	0.00	-	-
Years of publication	≥2020	9	101,674	20.88 (16.54, 25.21)	5,077.0	0.000	99.8
	<2020	9	46,817	20.91 (17.89, 23.94)	405.69	0.000	98.2
Sample size	≥1,081	9	143,763	15.58 (11.24, 19.92)	5,473.05	0.000	99.9
	<1,081	9	4,728	27.61 (18.23, 37.00)	473.6	0.000	98.5
Risk of bias	Low	8	61,713	26.08 (18.86, 33.29)	6,428.29	0.00	99.9
​	Moderate	10	86,778	23.98 (16.67, 29.29)	1772.13	0.00	99.5

CI, Confidence Interval; SNNPR, Southern nation nationality and people region.

### Commonest Types of Congenital Anomalies Among Newborns

Of the total neonatal anomalies identified, central nervous system abnormality was the commonest type occurring in 48.7%, followed by anomalies with more than one system involvement (14.9%) (Table 3).

**TABLE 3:** Types of congenital anomalies among newborns (Ethiopia, 2025).

S. No	Types of congenital anomalies	Frequency (%)
1	Central nervous anomalies	1,099 (48.7)
2	Musculoskeletal anomalies	194 (8.6)
3	Gastro intestinal tract anomalies	125 (5.5)
4	Genitourinary system anomalies	56 (2.5)
5	Down syndrome/genetic anomalies	24 (1.1)
6	Orofacial anomalies and head, and face malformation	222 (9.8)
7	Anomalies with more than one system involvement	99 (4.4)
8	Undefined anomalies	391 (17.3)
9	CVS anomalies	48 (2.10)

CVS, Cardiovascular system.

### Sensitivity Analysis

Sensitivity analysis was performed to evaluate the effect of each study on the pooled prevalence of congenital anomalies among newborns by using the leave-one-out approach, excluding each study step-by-step from the analysis. The results showed that there was no a single study that significantly affects the overall prevalence of congenital anomalies among newborns (Supplementary File 2).

### Publication Bias

The graphical inspection of the funnel plot was symmetrical in distribution indicating the absence of publication bias among the included studies. Egger’s (p = 0.769) and Begg’s test (p = 0.386) computed to prove the existence of publication bias, also revealed no evidence of publication bias among the included studies.

### Predictors of Congenital Anomalies Among Newborns in Ethiopia

Data regarding 25 identified predictors of congenital anomalies among newborns were extracted from the available studies and the results pooled odds ratio for each identified variable were presented by forest plot (Supplementary File 3). For each identified predictors, sensitivity analysis was also carried out by excluding each study one by one, but the result showed that there was no strong evidence for the effect of a single study on the overall results. Moreover, for each identified variable publication bias was assessed and no publication bias was detected. Pooled odds ratio (POR), level of significance, heterogeneity, and publication bias for each identified variable were summarized below (Table 4).

**TABLE 4:** Summary of the predictors of congenital anomalies among newborns (Ethiopia, 2025).

S. no	Identified factors	No. of studies	POR (95%CI)	P-value	Heterogeneity		Publication bias (Egger’s test)
					I^2^	p-value	
1	Age of the mother (35 years and above)	8	3.29 (1.59, 6.82)	0.001^a^	88.9	<0.001	0.362
2	Sex of newborns (male)	10	0.98 (0.74, 1.31)	0.886	69.8	<0.001	0.196
3	Urban residence	7	0.74 (0.47, 1.16)	0.188	80.5	<0.001	0.190
4	Marital status (married)	4	0.83 (0.36, 1.92)	0.670	85.4	<0.001	0.981
5	No formal education among mothers^b^	4	1.35 (1.12, 1.63)	0.002^a^	16.3	0.310	0.450
6	Gravidity (Primigravida)^b^	5	0.98 (0.79, 1.21)	0.817	45.5	0.119	0.810
7	Current or former maternal cigarate smoking	6	2.98 (1.56, 5.67)	0.001^a^	71.1	0.004	0.439
8	Maternal alcoholic drinking habit	7	2.66 (1.28, 5.51)	0.009^a^	86.8	<0.001	0.766
9	Chat chewing	4	3.37 (1.57, 7.21)	0.002^a^	85.9	<0.001	0.656
10	Absence of folic acid supplementation	7	4.29 (2.35, 7.83)	<0.001^a^	87.8	<0.001	0.356
11	Exposure to pesticide^b^	3	3.23 (2.02, 5.16)	<0.001^a^	0.00	0.486	0.179
12	Maternal X-ray exposure^b^	2	2.51 (0.91, 6.95)	0.076	0.00	0.379	0.303
13	Use of family planning before pregnancy	2	1.14 (0.47, 2.76)	0.771	86.0	0.008	0.243
14	Use of medication during pregnancy	6	4.37 (2.11, 9.06)	<0.001^a^	90.2	<0.001	0.435
15	History of maternal chronic illness	6	3.76 (1.72, 8.20)	0.001^a^	89.9	<0.001	0.430
16	History of CA in the former child	3	2.15 (0.92, 5.03)	0.079	68.6	0.041	0.534
17	Family history of CA	3	1.73 (0.79, 3.81)	0.171	65.7	0.045	0.058
18	History of abortion^b^	4	1.23 (0.90, 1.68)	0.202	0.00	0.866	0.721
19	History of stillbirth^b^	3	1.21 (0.81, 1.79)	0.355	0.00	0.762	0.910
20	Absence of ANC follow-up	6	1.50 (0.61, 3.71)	0.377	94.2	<0.001	0.791
21	Being preterm	6	2.35 (1.59, 3.47)	<0.001^a^	50.1	0.075	0.109
22	Other than vaginal delivery	3	0.72 (0.29, 1.81)	0.482	84.1	0.002	0.296
23	Multiple pregnancy	5	3.66 (1.99, 6.71)	<0.001^a^	71.3	0.007	0.150
24	Being low birth weight during delivery^b^	6	5.46 (4.41, 6.75)	<0.001^a^	46.1	0.098	0.156
25	Birth order (being second order or more)	3	1.05 (0.63, 1.75)	0.851	67.0	0.048	0.967

^a^ Significant association; CA, Congenital anomalies.

^b^ Fixed effect model employed.

## Discussion

This systematic review and meta-analysis was conducted to estimate the pooled prevalence of congenital anomalies and its predictors among newborns in Ethiopia. The reviewed results revealed that the pooled prevalence of congenital anomalies among newborns was found to be 21.25 (95% CI: 17.93–24.58). The finding is comparable with the results of the studies conducted in India 23.1 per 10,000 [34], and Iran 23.0 per 1,000 [35]. However, prevalence of congenital anomalies among newborns in this review was higher than the finding of the studies conducted in Nepal 4.2 per 1,000 [36], China 15.6 per 1,000 [37], Brazil 16 per 1,000 [38], and Tanzania 2.8 per 1,000 [39]. The observed variations in the prevalence of congenital anomalies among newborns may be attributed to differences in socio-demographic and cultural characteristics, national income levels, the quality of healthcare services, and the health-seeking behaviors of communities.

Congenital anomalies affecting the central nervous system were the most frequently reported, followed by those with unspecified system involvement, and whereas Down syndrome was the least commonly observed. Comparable findings were detected by the studies conducted in China [37], and Tanzania [39]. However, the results of the studies conducted in Iran [35] and India [40] indicated that anomalies of musculoskeletal system and genitourinary system were the most frequent types. This variation may be attributed to differences in study settings; some studies were conducted in referral facilities where patients with advanced or complicated cases were admitted for specialized care and management, while others included all types of healthcare facilities providing labor and delivery services.

It was also found that the age of the mother, educational status of the mothers, habit of cigarate smoking (current or former), alcohol drinking, chat chewing, absence of folic acid supplementation during pregnancy, history of exposure to pesticide, use of medication during pregnancy, history of maternal chronic illness, preterm delivery, multiple pregnancy, and low birth weight (<2.5 kg) were significantly associated with congenital anomalies.

Unlike the studies conducted in Saudi Arabia [41], and Tanzania [42], our findings demonstrated a significant association between maternal age and congenital anomalies, with mothers over the age of 35 being more likely to give birth with congenital anomalies compared to those under 35 years of age. This is also supported by the studies conducted in Kenya [43], Egypt [44], and, Hungary [45]. These findings suggest that increasing maternal age may be one of the non-modifiable risk factors for congenital anomalies in humans. This association could be explained by the greater likelihood of chromosomal meiotic errors and prolonged exposure to environmental toxins over time in older mothers [45, 46]. Additionally, the higher incidence of aneuploidy and the increased prevalence of age-related comorbidities such as diabetes, hypertension, and metabolic syndrome may further contribute to this risk explanation [47].

The findings of this study indicated a statistically significant association between certain maternal behavioral characteristics such as smoking, alcohol consumption, khat chewing, pesticide exposure, and medication use during pregnancy and the occurrence of congenital anomalies. This may be attributed to the teratogenic effects of these substances, which can cross the placental barrier and directly affect fetal tissues during organogenesis, potentially leading to structural abnormalities. This is because of the fetal tissues are not matured; they are highly susceptible to the adverse effects of these substances [48, 49]. For instance, a recent experimental study demonstrated that khat could disrupt intrauterine development by reducing fetal lipid availability, thereby altering the biological structure of fetal organs [16].

Absence of folic acid supplementation during pregnancy was also found to be significantly associated with the occurrence of congenital anomalies. This association is well-supported, as numerous studies have shown that folic acid plays a crucial role in the synthesis of essential biomolecules necessary for cell growth, division, and differentiation processes that are particularly vital during fetal development [50–53].

Our findings also revealed a significant association between low birth weight and the presence of congenital anomalies, which is consistent with studies conducted in various regions worldwide [54–56]. This relationship may be explained by shared prenatal factors that both hinder fetal growth and elevate the risk of structural or functional abnormalities. Additionally, certain congenital anomalies may themselves cause intrauterine growth restriction, leading to low birth weight [57]. However, a retrospective study conducted in Tanzania reported that a birth weight of ≥2.5 kg was significantly associated with congenital anomalies [42]. This discrepancy could be attributed to methodological differences or variations in maternal and fetal health conditions.

Consistent with studies conducted in China [55], India [58], and USA [59], the present study also found a significant association between preterm delivery and congenital anomalies. Our results indicate that preterm infants are more likely to be born with congenital anomalies compared to those delivered at full term. This association may be explained by at least two potential pathways. In some instances, shared risk factors such as maternal smoking or obesity may independently contribute to both preterm birth and congenital anomalies. In other cases, specific factors, such as exposure to valproic acid or insufficient periconceptional folic acid intake, may directly lead to congenital anomalies like spinal bifida, which in turn increases the risk of preterm birth [59, 60].

### Conclusion and Recommendation

More than two out of every one hundred fetuses were born with congenital anomalies. Behavioral characteristics of mother such as cigarette smoking, alcohol consumption, pesticide exposure, use of medications during pregnancy, and lack of folic acid supplementation during pregnancy were significantly associated with congenital abnormalities among newborns in Ethiopia. Additionally, a history of maternal chronic illness, twin pregnancy, and low birth weight (<2.5 kg) were also significantly linked to the occurrence of congenital anomalies.

Therefore, in order to combat congenital anomalies, healthcare providers in collaboration with relevant stakeholders should focus on strengthening pre-conceptional folate programs, propose strict agricultural protection policies for pregnant women, reducing substance use and inappropriate medication during pregnancy.

### Limitations of the Study

This systematic review and meta-analysis has some limitations that need to be considered. Most of the studies included the reviews were cross-sectional. As a result, a cause-effect relationship cannot be ascertained. Moreover, there was substantial heterogeneity among the included studies and the cause/s for the heterogeneity thus remains undetected. Some of Ethiopian regions were underrepresented, with certain areas having only one study or none at all. This may reduce generalizability of the study to the entire country. Exclusion of studies with poor quality score may also under/or overestimate the prevalence of congenital anomalies. Variations in reporting practices, diagnostic skills and equipment in different facilities may have contributed to misclassification bias or underreporting of certain types of congenital anomalies.

## Data Availability

The original contributions presented in the study are included in the article/Supplementary Material; further inquiries can be directed to the corresponding author.
