# Supplementary material for: A Silent Epidemic of Congenital Anomalies and Its Predictors Among Newborns in Ethiopia: A Systematic Review and Meta-Analysis
Source: Public Health Rev. 2026 Feb 23;47:1608833. doi: 10.3389/phrs.2026.1608833 (PMC12968043; doi:10.3389/phrs.2026.1608833)
Supplement: Supplementary file 1 [file Supplementaryfile1.docx]

**Supplementary file 1**: Searching strategies for systematic review and meta-analysis of congenital anomalies among newborns in Ethiopia

| **Data based used** | **Search Item** |
| --- | --- |
| PubMed | (((((((((((Prevalence) OR (Magnitude)) AND (congenital anomalies))  OR (birth defects)) OR (congenital malformations)) OR (congenital abnormalities)) AND (associated factors)) OR (predictors)) OR (determinant)) AND (newborns)) OR (infants)) OR (neonates)) AND (Ethiopia)) |
| Scopus | ( ALL (congenital anomalies OR congenital abnormalities OR "  Birth defect" OR "congenital malformations " AND  TITLE-ABS-KEY (newborns OR neonates OR infants AND "Ethiopia") |
| Web of Science | ((congenital OR "congenital anomaly*" OR "congenital defect*" OR "birth defect*" OR "birth anomaly*" OR "birth abnormalities" OR "congenital malformation*") AND (newborn* OR neonate* OR infant* OR "new born" OR "new-born") AND (Ethiopia OR Ethiopian)) |
| Wiley Online Library | ("congenital anomaly*" OR "congenital defect*" OR "birth defect*" OR "congenital malformation*") AND (newborn* OR neonate* OR infant*)  AND (Ethiopia OR Ethiopian) |
| Google scholar | “Congenital anomalies OR congenital abnormalities OR congenital malformation OR birth defects OR” AND “newborns OR neonate” |
