# Supplementary material for: A Silent Epidemic of Congenital Anomalies and Its Predictors Among Newborns in Ethiopia: A Systematic Review and Meta-Analysis
Source: Public Health Rev. 2026 Feb 23;47:1608833. doi: 10.3389/phrs.2026.1608833 (PMC12968043; doi:10.3389/phrs.2026.1608833)

**Suplementary file 3: Predictors of congenital anomalies among newborns in Ethiopia**

**Maternal age of 35 and above during pregnancy**


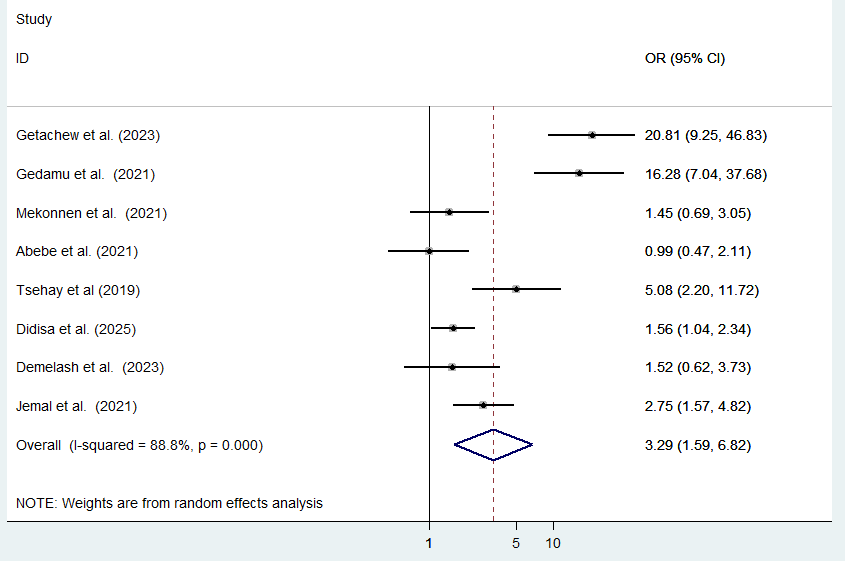


**Being male sex**


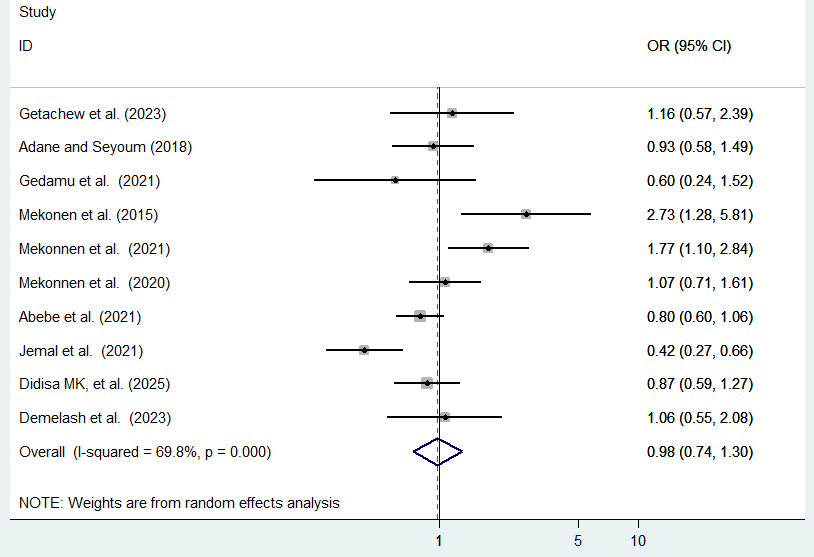


**Urban residence**

**
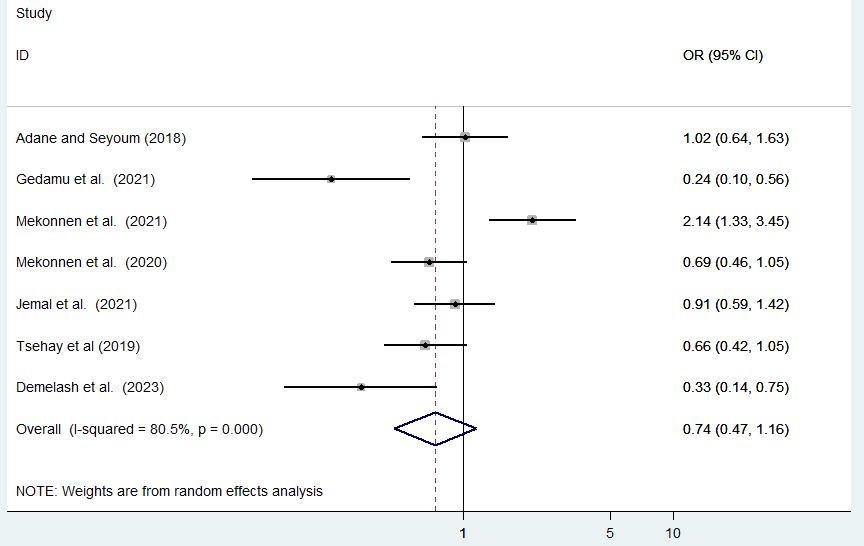
**

**Being married as compared with others**
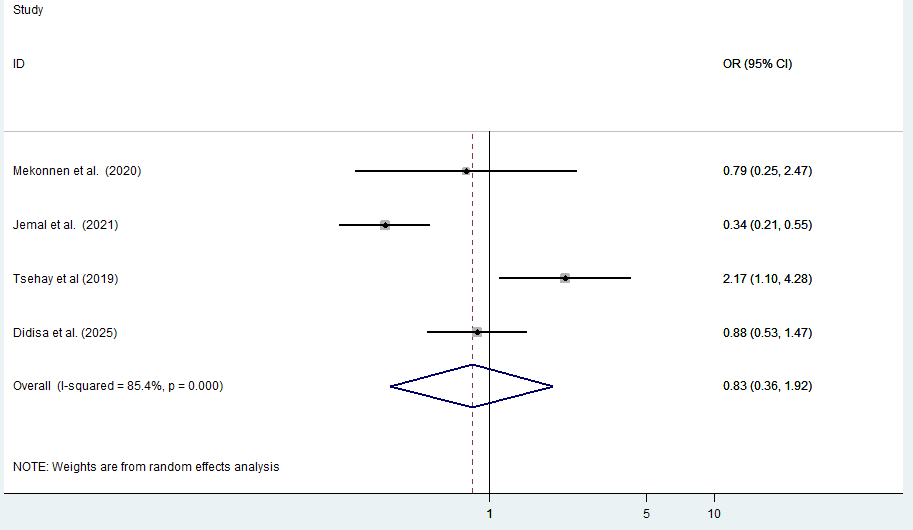


**No formal education of the mother**

**
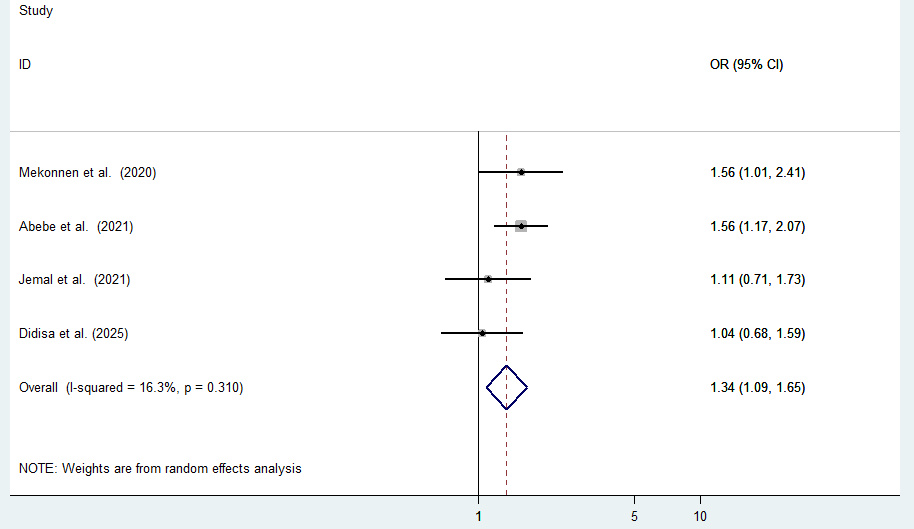
**

**Primigravidity**


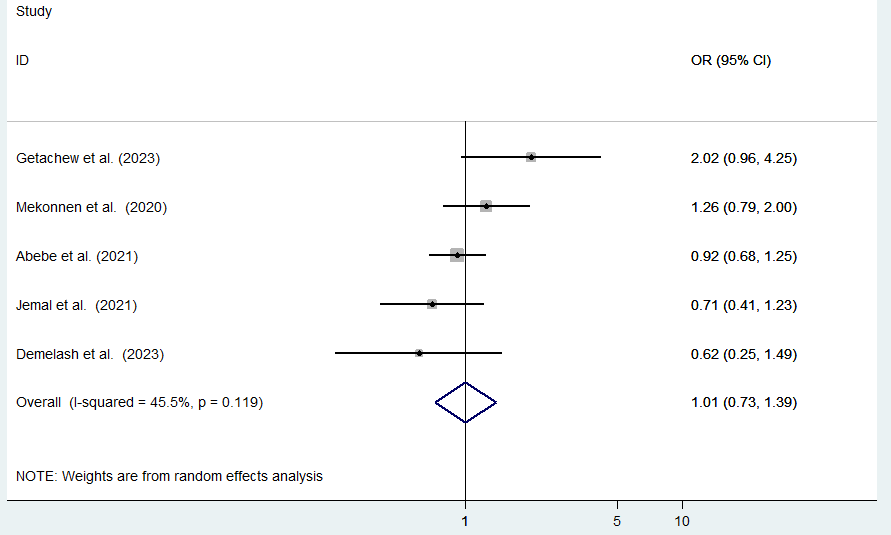


**Being current or former smoker**

**
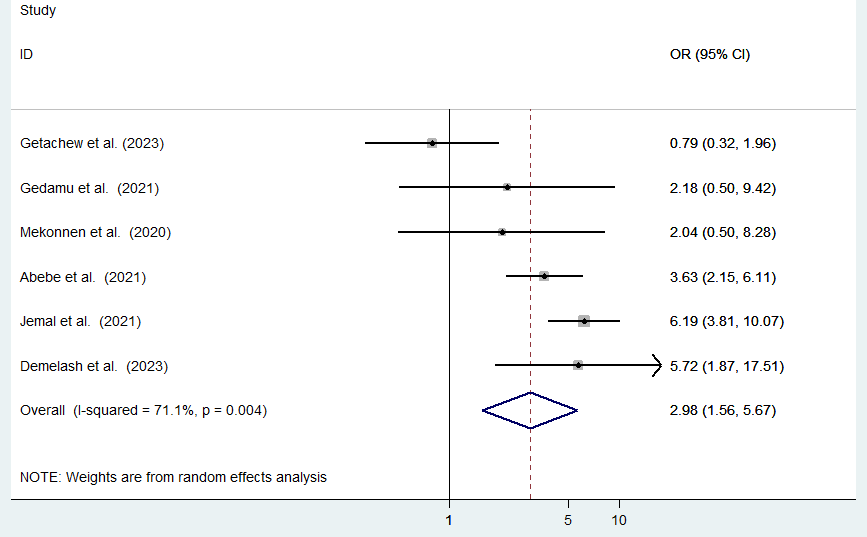
**

**Habit of maternal alcoholic drinking during pregnancy**


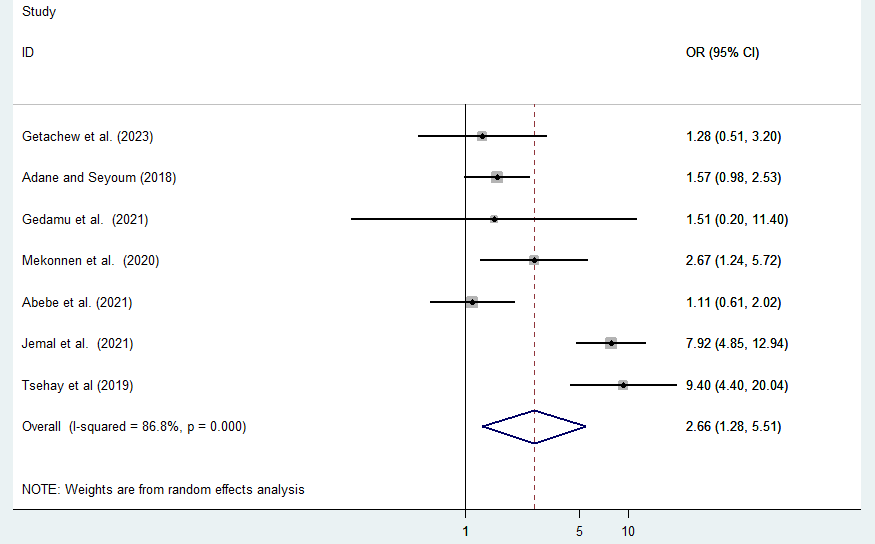


**Habits of maternal chat chat chewing during pregnancy**

**
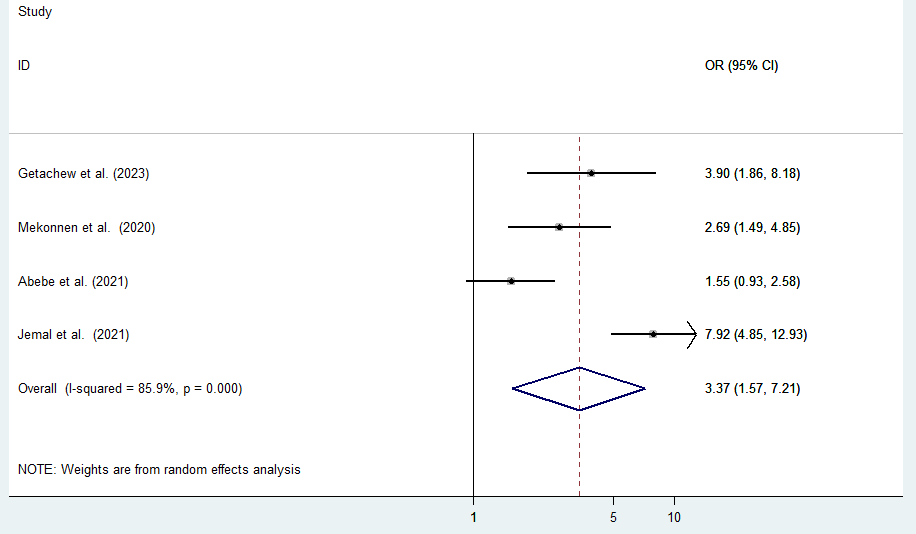
**

**Absence of folic acid supplementation during pregnancy**


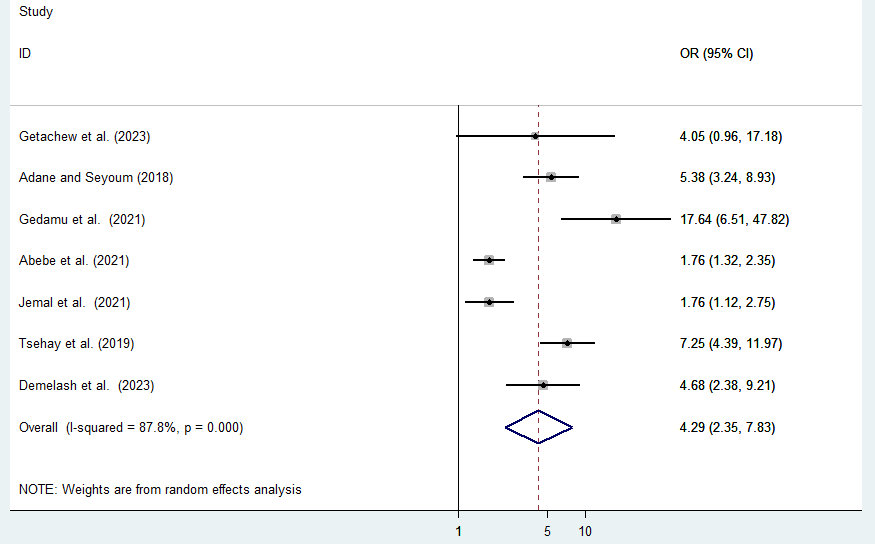


**Exposure to pesticide during pregnancy**

**
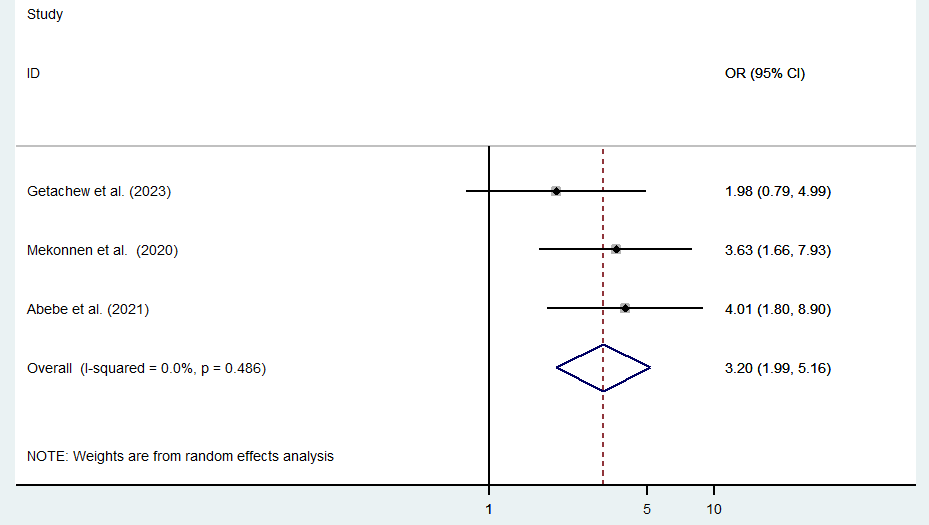
**

**Maternal X-ray exposure during pregnancy**


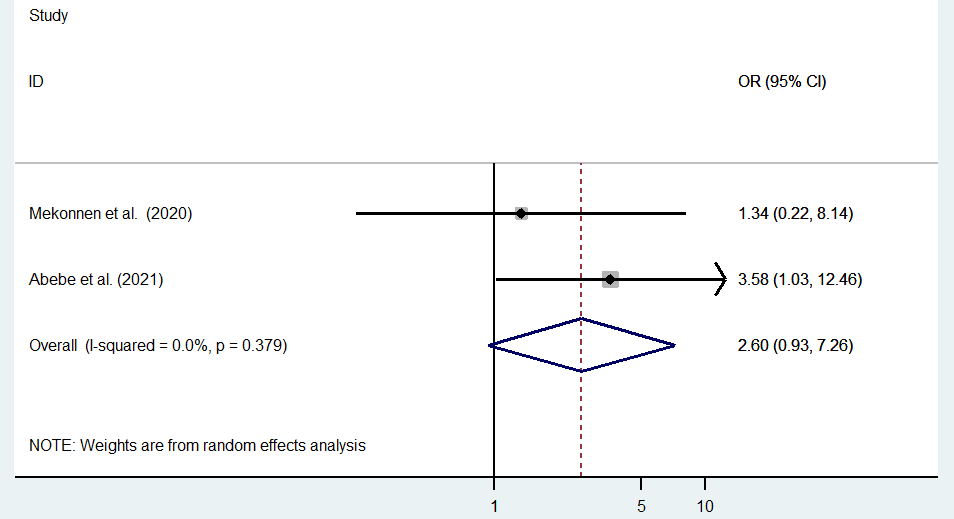


**Use of FP before current pregnancy**

**
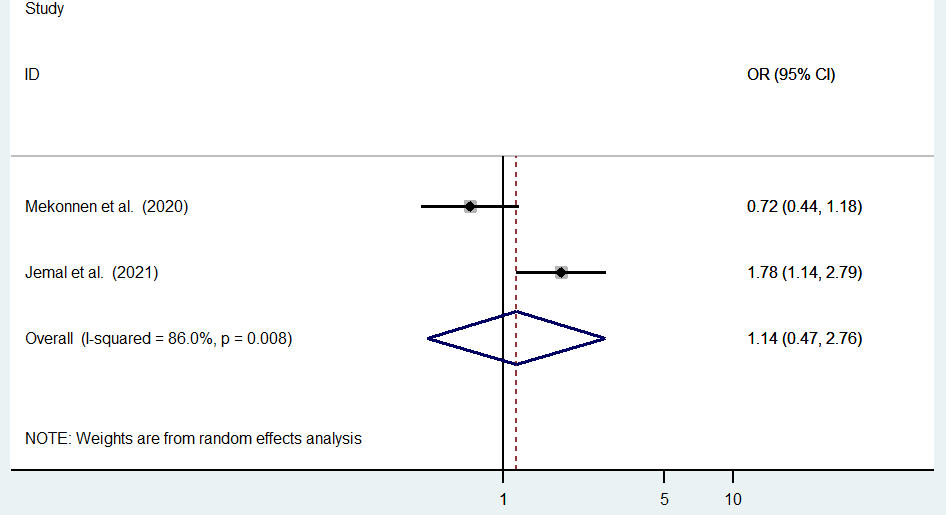
**

**Use of medication during pregnancy**

**
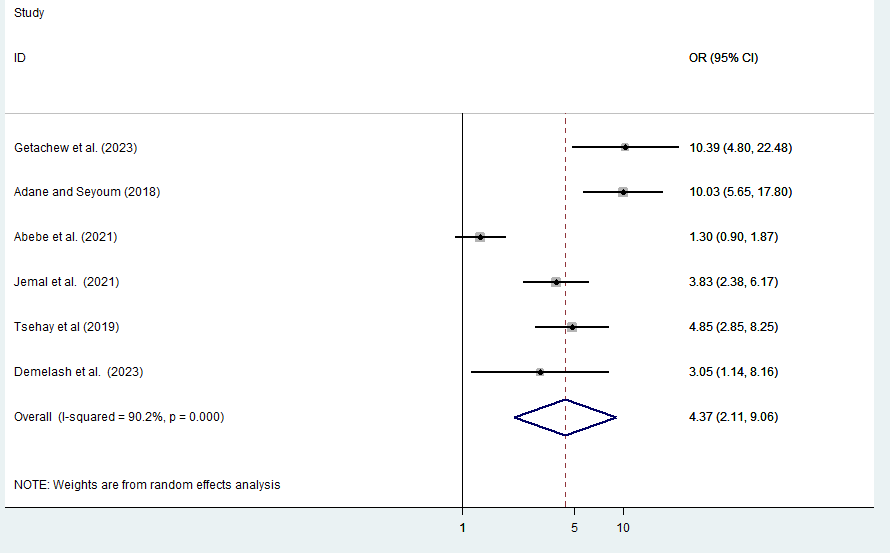
**

**Maternal chronic illness**

**
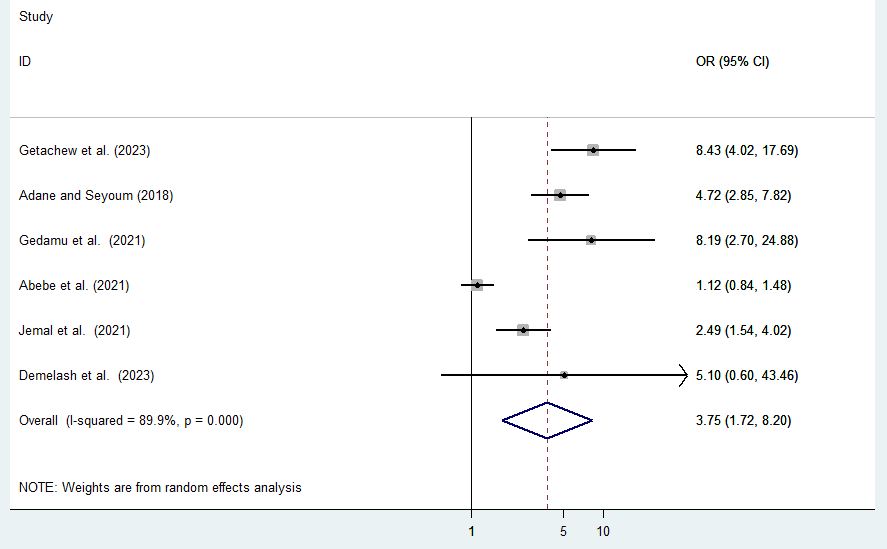
**

**History of previous congenital anomalies**

**
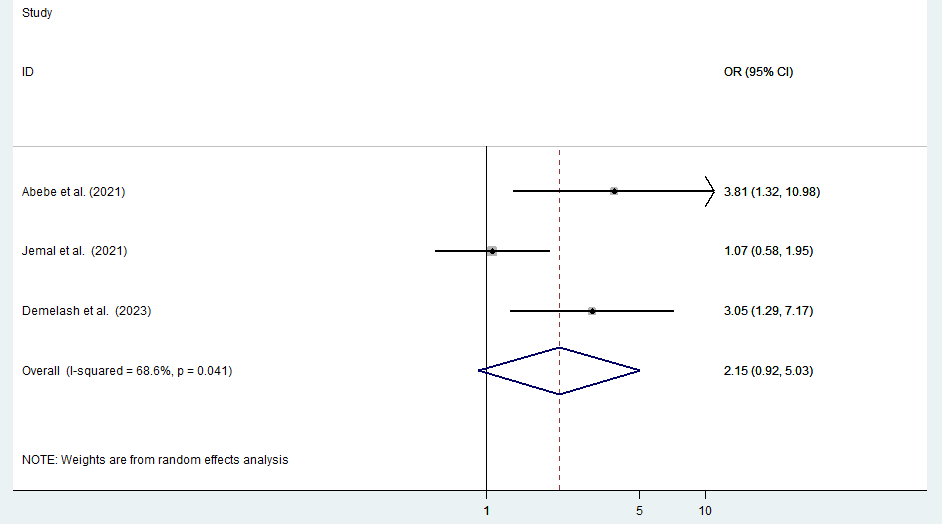
**

**Family history of congenital anomalies**

**
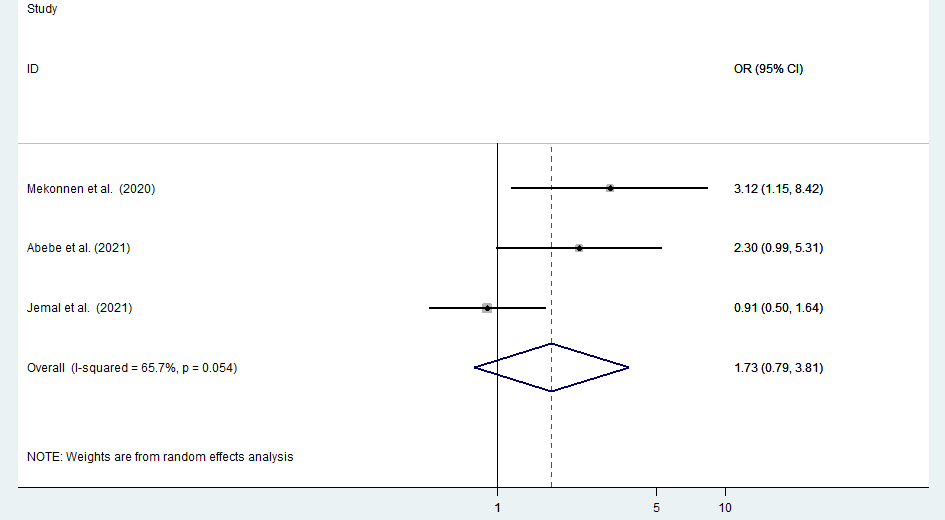
**

**Maternal history of abortion**

**
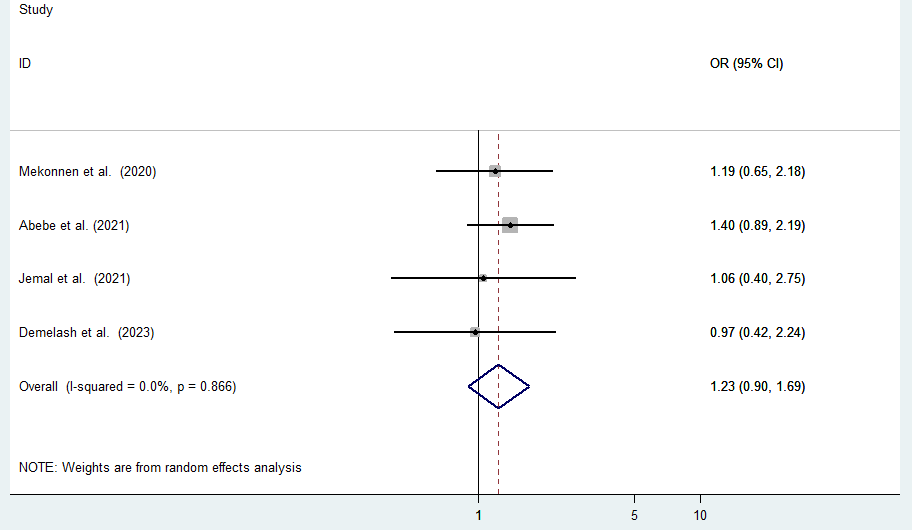
**

**Maternal history of still birth**

**
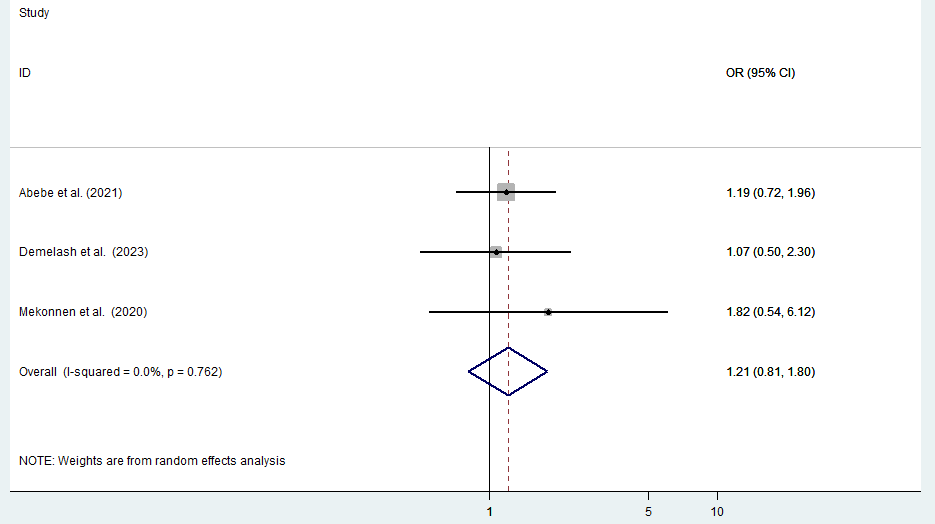
**

**Absence of ANC Visit**

**
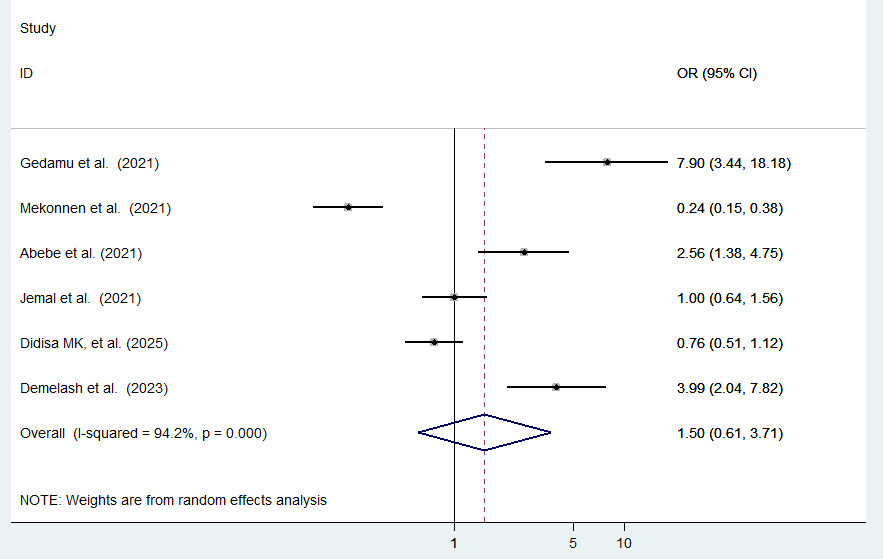
**

**Being preterm**

**
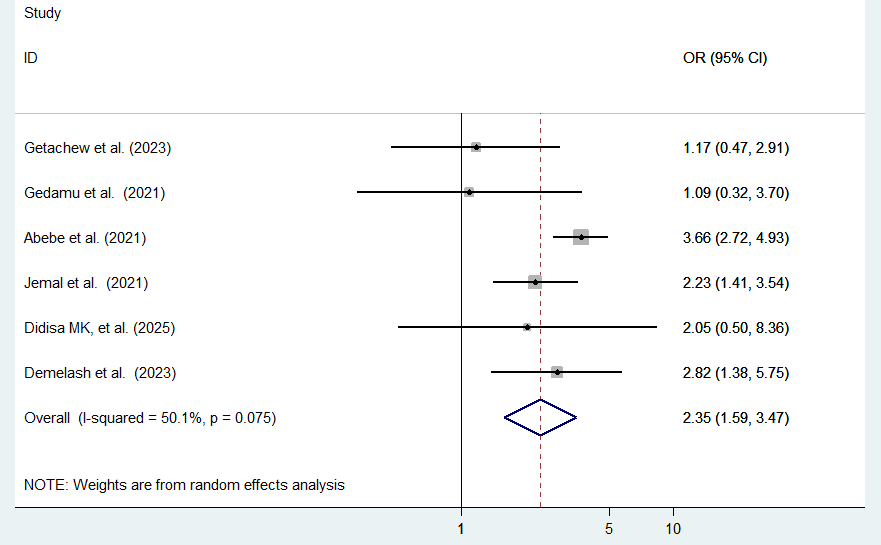
**

**Mode of delivery (CS)**

**
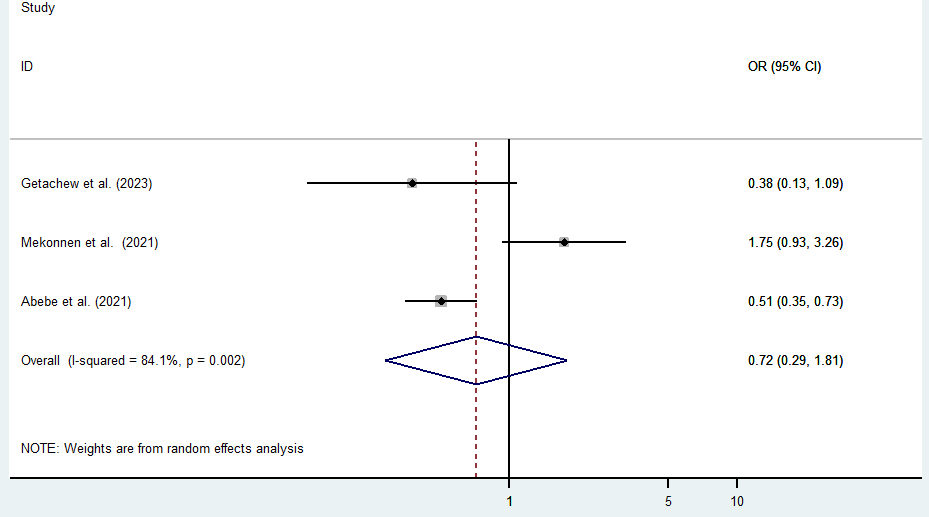
**

**Multiple pregnancy type**

**
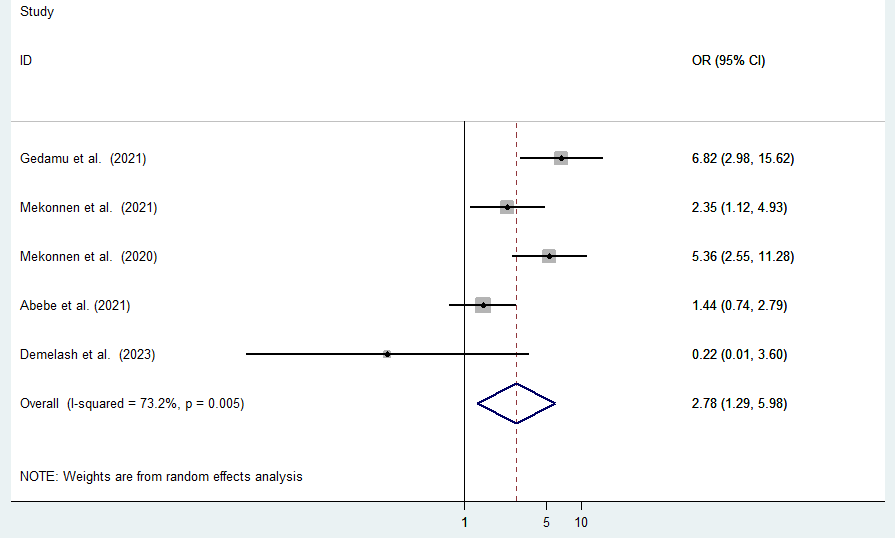
**

**Low birth weight**

**
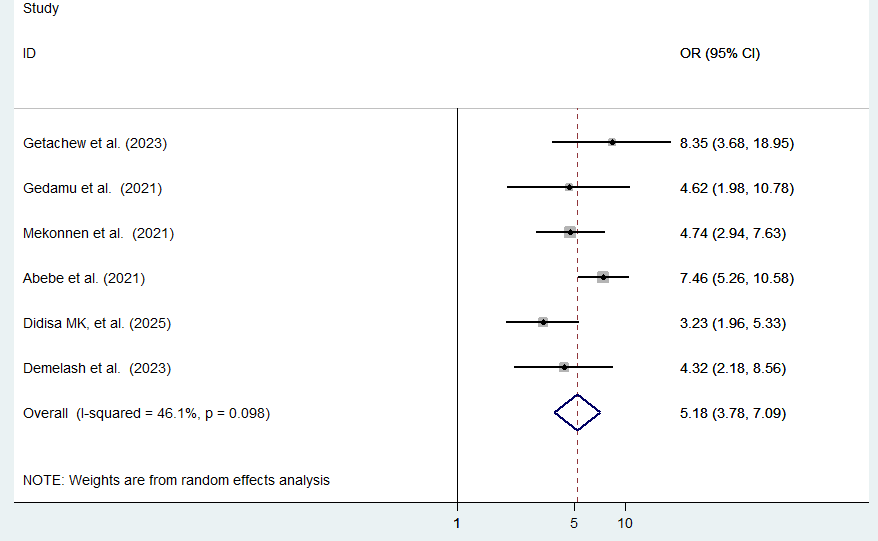
**

**Being second or more birth order**


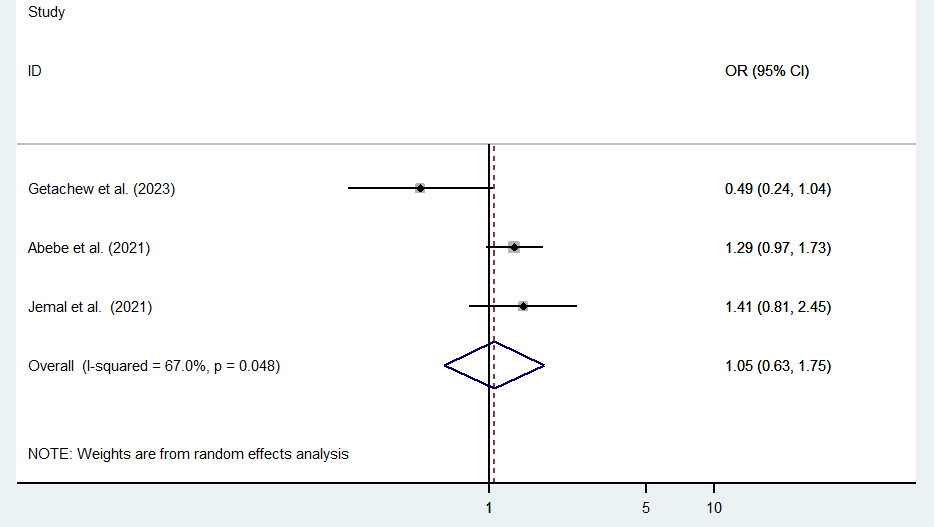

Supplement: Supplementary file 2 [file Supplementaryfile3.docx]
