# Supplementary material for: A Silent Epidemic of Congenital Anomalies and Its Predictors Among Newborns in Ethiopia: A Systematic Review and Meta-Analysis
Source: Public Health Rev. 2026 Feb 23;47:1608833. doi: 10.3389/phrs.2026.1608833 (PMC12968043; doi:10.3389/phrs.2026.1608833)

Supplementary file 2: Results of sensitivity tests for the estimate of pooled prevalence of congenital anomalies among the newborns in Ethiopia


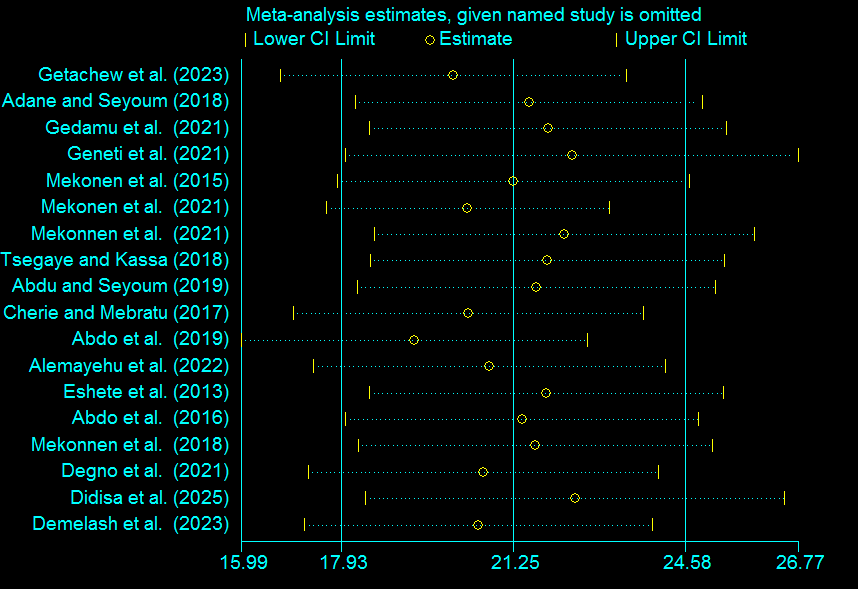

Supplement: Supplementary file 3 [file Supplementaryfile2.docx]
